# CT, US and MRI of xanthine urinary stones: in-vitro and in-vivo analyses

**DOI:** 10.1186/s12894-020-00736-w

**Published:** 2020-10-12

**Authors:** Stephanie B. Shamir, Qi Peng, Alan H. Schoenfeld, Beth A. Drzewiecki, Mark C. Liszewski

**Affiliations:** 1 Department of Radiology, Montefiore Medical Center, Albert Einstein College of Medicine, 111 East, 210th Street, Bronx, NY 10467 USA; 2 Division of Pediatric Urology, Department of Urology, Montefiore Medical Center, Albert Einstein College of Medicine, 3400 Bainbridge Ave, 5th Floor, Bronx, NY 10467 USA; 3 Division of Pediatric Radiology, Department of Radiology, Montefiore Medical Center, Albert Einstein College of Medicine, 111 East, 210th Street, Bronx, NY 10467 USA

## Abstract

**Background:**

Xanthine urinary stones are a rare entity that may occur in patients with Lesch–Nyhan syndrome receiving allopurinol. There is little literature describing imaging characteristics of these stones, and the most appropriate approach to imaging these stones is therefore unclear. We performed in-vitro and in-vivo analyses of xanthine stones using computed tomography (CT) at different energy levels, ultrasound (US), and magnetic resonance imaging (MRI).

**Methods:**

Five pure xanthine stones from a child with Lesch-Nyhan were imaged in-vitro and in-vivo. CT of the stones was performed at 80 kVp, 100 kVp, 120 kVp and 140 kVp and CT numbers of the stones were recorded in Hounsfield units (HU). US of the stones was performed and echogenicity, acoustic shadowing and twinkle artifact were assessed. MRI of the stones was performed and included T2-weighted, ultrashort echo-time-weighted and T2/T1-weighted 3D bFFE sequences and signal was assessed.

**Results:**

In-vitro analysis on CT demonstrated that xanthine stones were radiodense and the average attenuation coefficient did not differ with varying kVp, measuring 331.0 ± 51.7 HU at 80 kVp, 321.4 ± 63.4 HU at 100 kVp, 329.7 ± 54.2 HU at 120 kVp and 328.4 ± 61.1 HU at 140 kVp. In-vivo analysis on CT resulted in an average attenuation of 354 ± 35 HU. On US, xanthine stones where echogenic with acoustic shadowing and twinkle artifact. On MRI, stones lacked signal on all tested sequences.

**Conclusion:**

Xanthine stone analyses, both in-vitro and in-vivo, demonstrate imaging characteristics typical of most urinary stones: dense on CT, echogenic on US, and lacking signal on MRI. Therefore, the approach to imaging xanthine stones should be comparable to that of other urinary stones.

## Background

Xanthine urolithiasis is a rare entity, occurring in patients with Lesch–Nyhan syndrome, who are receiving allopurinol treatment, and in patients with hereditary xanthinuria [1–4]. While a rare condition, xanthine urolithiasis may cause recurrent symptoms in this group of patients and require frequent medical attention. Children with Lesch–Nyhan syndrome are developmentally delayed, and often cannot appropriately verbalize their symptoms or localize their pain, making clinical assessment difficult. This often leads to multiple imaging studies over time. Understanding the imaging characteristics of these stones on different imaging modalities is imperative for effective clinical management.

Little is written in the medical literature about the imaging of xanthine stones, and no previous in-vitro studies have described the imaging characteristics of xanthine stones on CT, US or magnetic resonance imaging (MRI). A small number of in-vivo studies have described xanthine stones as radiolucent on radiographs and excretory urograms, as echogenic with posterior shadowing on ultrasound (US) and as having a computed tomography (CT) number ranging from 276–480 HU on conventional single energy CT [1–4].

Given the small number of previous studies on the topic, we sought to further characterize the imaging features of this rare urinary stone. Utilizing an in-vitro and in-vivo study design we characterized the imaging findings of xanthine stones on varying CT energy levels (80, 100, 120 and 140 kVp), US and MRI.

## Methods

This is a retrospective study, and for this type of study formal consent and an ethics approval by an IRB are not required.

Stones that were previously passed by a child aged 3–10 years old with Lesch–Nyhan syndrome on allopurinol therapy were retained and analyzed. Stones’ diameters measured 4.2 mm, 3.0 mm, 3.9 mm, 2.2 mm and 2.6 mm. The stones were then characterized on the available imaging modalities performed on the patient prior to the stones being passed.

In-vitro CT images of the stones were obtained using a GE LightSpeed VCT CT scanner. Stones were placed within saline-containing syringes arranged concentrically in an acrylic PMMA phantom. A 16 cm diameter CTDI phantom was used. CT imaging was performed at 80 kVp, 100 kVp, 120 kVp and 140 kVp levels. Tube current was 250 mA for 80 kVp, 100 kVp, and 120 kVp, and tube current was 210 mA at 140 kVp. Other imaging parameters included: 1.00 s scan time, 0.625 mm section thickness, 40 mm collimation, helical mode with 0.984 pitch, display Field of View (FOV) = 36 cm; scan FOV = 50 cm (adult). The corresponding CT numbers of the stones were recorded in Hounsfield units (HU) using region-of-interest sampling.

In-vivo CT images of the stones were obtained from two CT scans performed on the patient prior to stone passage. Both studies were performed at 100 kVp. The corresponding CT numbers of the stones were recorded in Hounsfield units (HU) using region-of-interest sampling.

US images of the stones were obtained using a General Electric (GE) Logiq E9 US machine. Stones were placed one by one in a 0.9% NaCl saline bath on a standoff pad in a round plastic container. Stones were imaged with 7 MHz and 15 MHz transducers. Imaging characteristics were recorded, including echogenicity of the stones, acoustic shadowing and twinkle artifact on color Doppler imaging.

In-vivo US images of the stones were obtained from three US scans performed on the patient prior to stone passage. Imaging characteristics were recorded, including echogenicity of the stones, acoustic shadowing and twinkle artifact on color Doppler imaging.

MR images of the stones were obtained on a 3 T Philips Achieva MRI scanner with a 32-channel head coil**.** Stones were first visualized in air-filled syringes and then in saline-filled syringes. T2-weighted (T2W) turbo spin echo (TSE) sequences, multi-slice balanced field echo (bFFE, or balanced steady-state free precession) sequences, and 3D stack-of-stars ultra-short-TE (UTE) sequences with radial k-space trajectory acquisition were tested on the phantoms [5]. TSE-based T2W sequences had repetition time (TR) = 1500 ms, echo time (TE) = 80 ms, flip angle = 90°, slice thickness = 4 mm, gap = 0.4 mm, matrix size = 156 × 126, and FOV = 200 × 174 mm; T2/T1-weighted bFFE sequences had TR = 2.7 ms, TE = 1.2, flip angle = 40°, thickness = 5 mm, gap = 1 mm, FOV = 256 × 260, matrix size = 144 × 163; and UTE sequence had TR = 4.27 ms, dual TE = 0.142/1.2 ms, Flip angle = 9°, FOV = 200 mm, matrix size = 184 × 184, number of excitations (NEX) = 1, slice thickness = 2.2 mm, and scan duration = 2 min 23 s. No in-vivo MRI images of the stones were performed on the patient prior to stone passage.

After imaging analysis of the stones was complete, chemical analysis of all stones was performed to confirm their composition.

## Results

Chemical analysis confirmed that all five stones where composed of 100% xanthine.

**Fig. 1:**
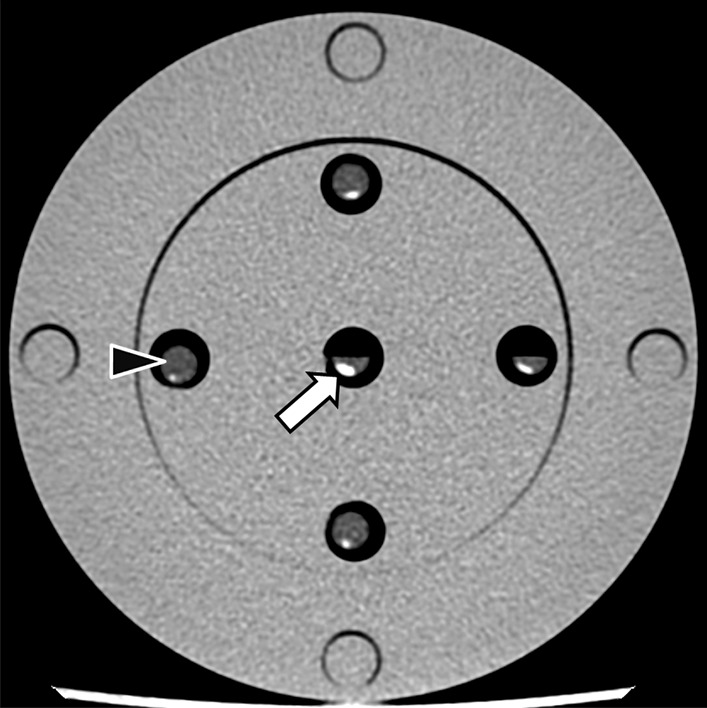
In vitro CT of xanthine urinary stones: axial CT image showing stones (arrow) within saline filled syringes (arrowhead) placed in an acrylic PMMA phantom

**Fig. 2:**
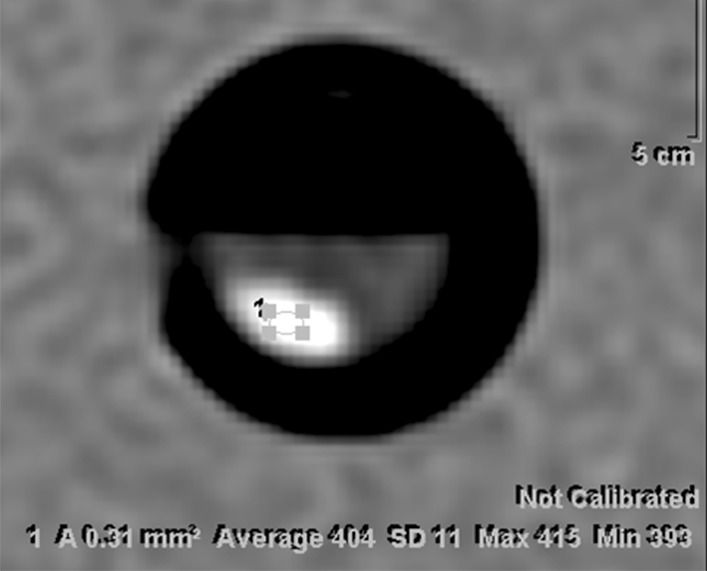
In vitro CT of xanthine urinary stones: magnified axial CT image showing measurement of CT number utilizing region of interest sampling

**Fig. 3:**
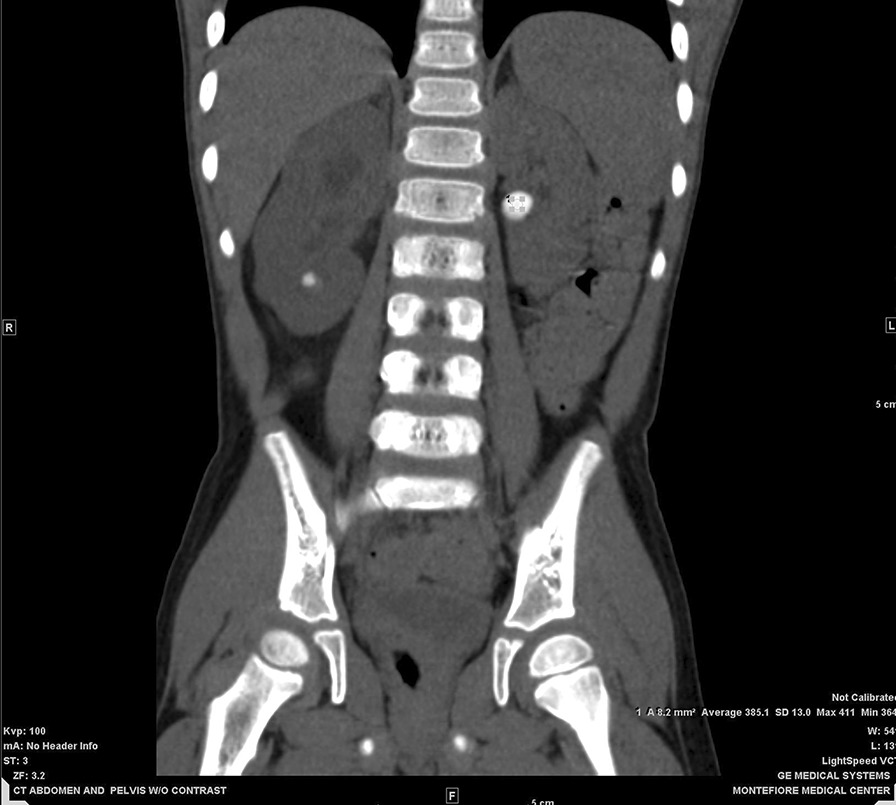
In vivo CT of xanthine urinary stones: coronal CT image showing a stone within the left kidney along with measurement of CT number utilizing region of interest sampling

On in-vitro CT analysis, stones had an average CT number of 331.0 ± 51.7 HU at 80 kVp, 321.4 ± 63.4 HU at 100 kVp, 329.7 ± 54.2 HU at 120 kVp and 328.4 ± 61.1 HU at 140 kVp (Figs. 1, 2) (Table 1) (Additional file 1). On in-vivo CT analysis performed on the patient prior to stone passage on two separate imaging studies at 100kVp, a 6 mm stone had an average CT number of 304 ± 26 HU, a 9 mm stone measured 374 ± 8 HU, and a 10 mm stone measured 383 ± 12 HU (Fig. 3).

**Table 1:** Xanthine urinary stone CT numbers at different energy levels

Energy level (kVp)	Mean CT number (HU)	Standard deviation	Maximum CT number (HU)	Minimum CT number (HU)
80	330.97	51.7	409	246
100	321.37	63.38	410.5	216.5
120	329.69	54.18	425	227
140	328.45	61.13	425.2	232

*HU *hounsfield unit

**Fig. 4:**
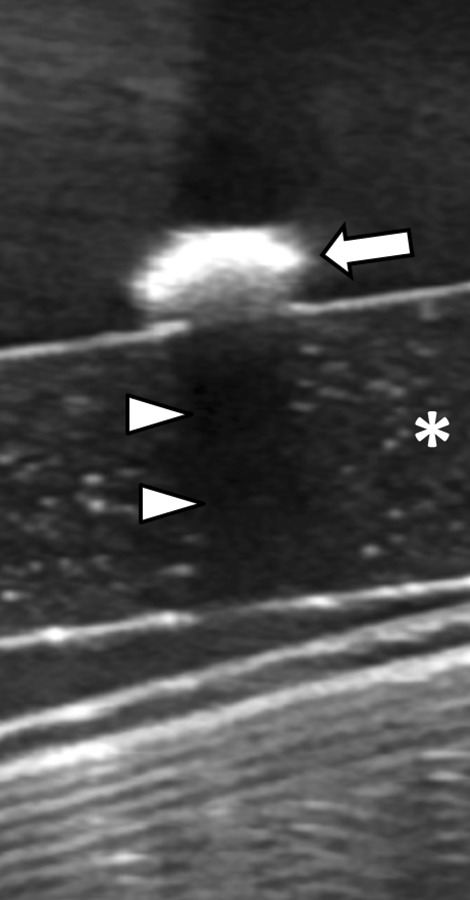
In vitro ultrasound of xanthine urinary stone: stone (arrow) is imaged in a water bath on a standoff pad (asterisk) and is echogenic with posterior acoustic shadowing (arrowheads)

**Fig. 5:**
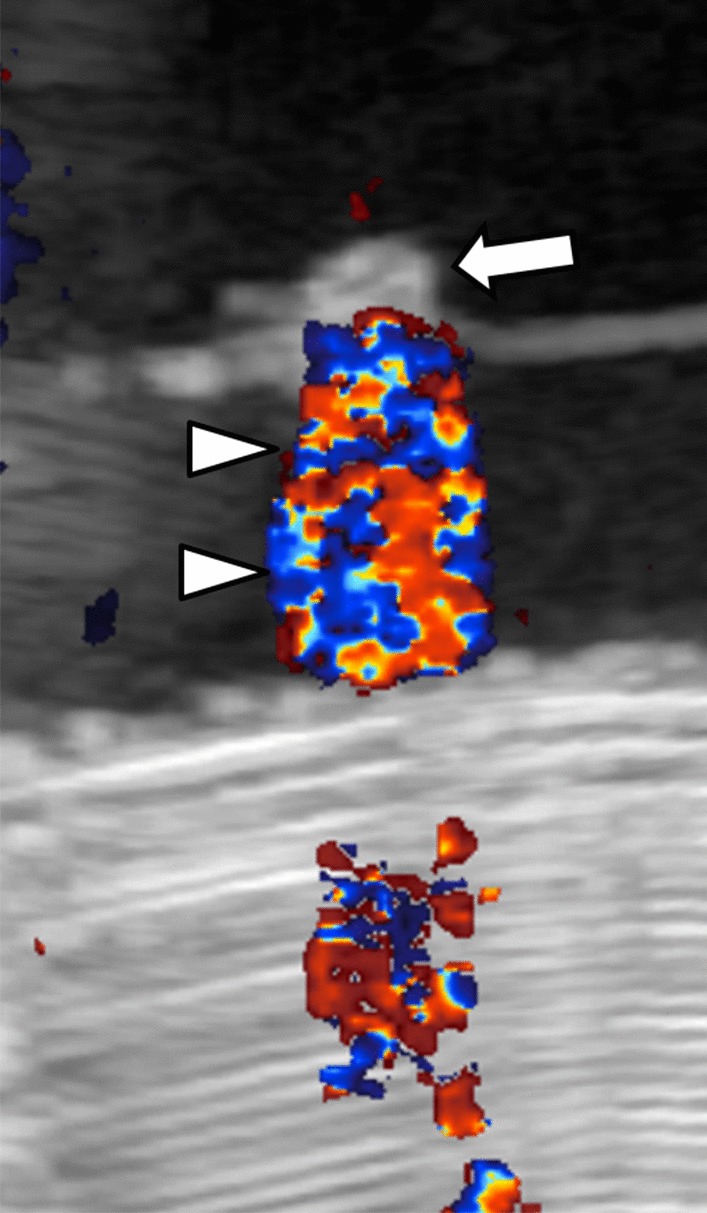
In vitro ultrasound of xanthine urinary stone: stone (arrow) shows posterior twinkle artifact (arrowheads) on color Doppler imaging

**Fig. 6:**
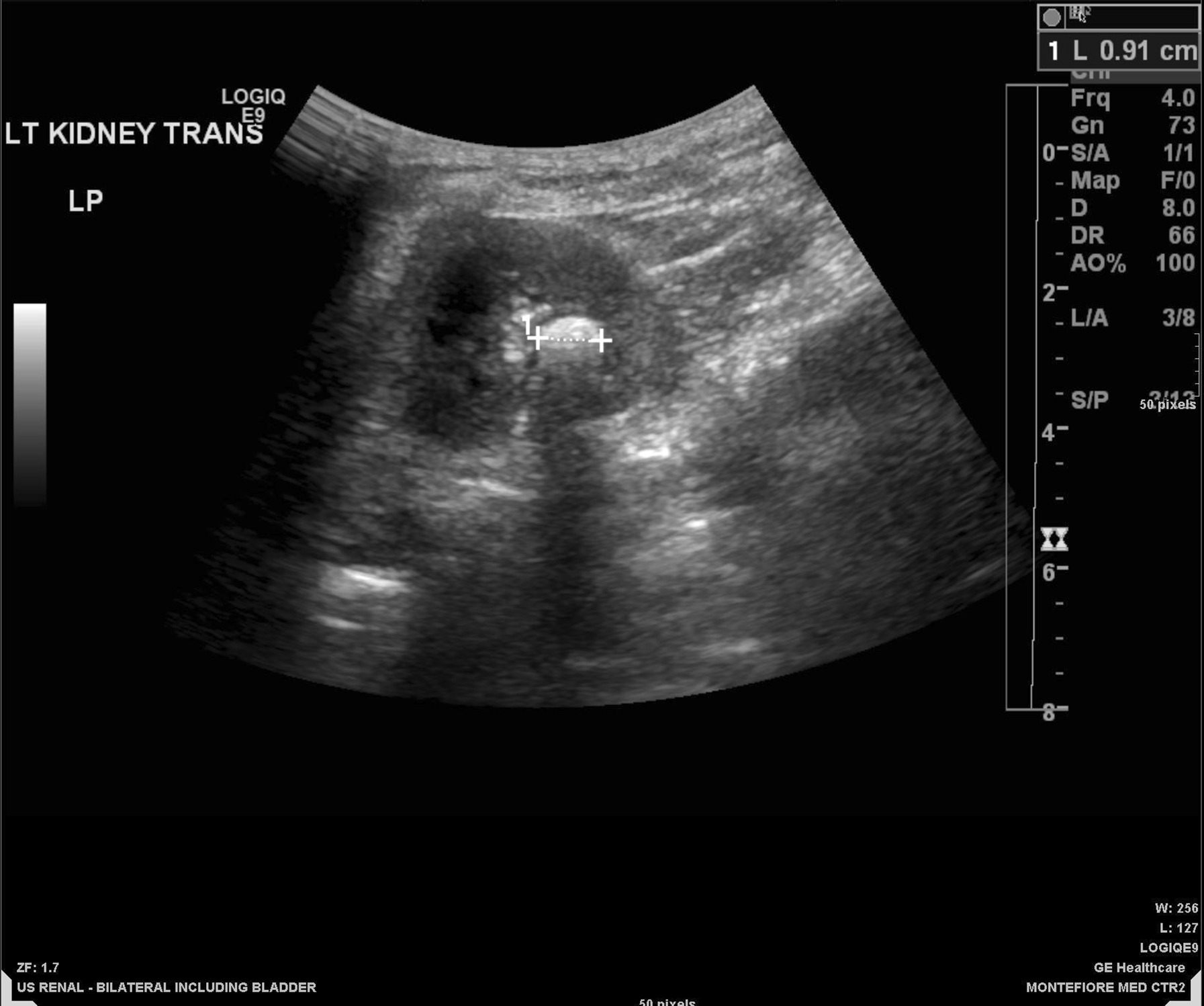
In vivo ultrasound of xanthine urinary stone: stone is echogenic with posterior acoustic shadowing

**Fig. 7:**
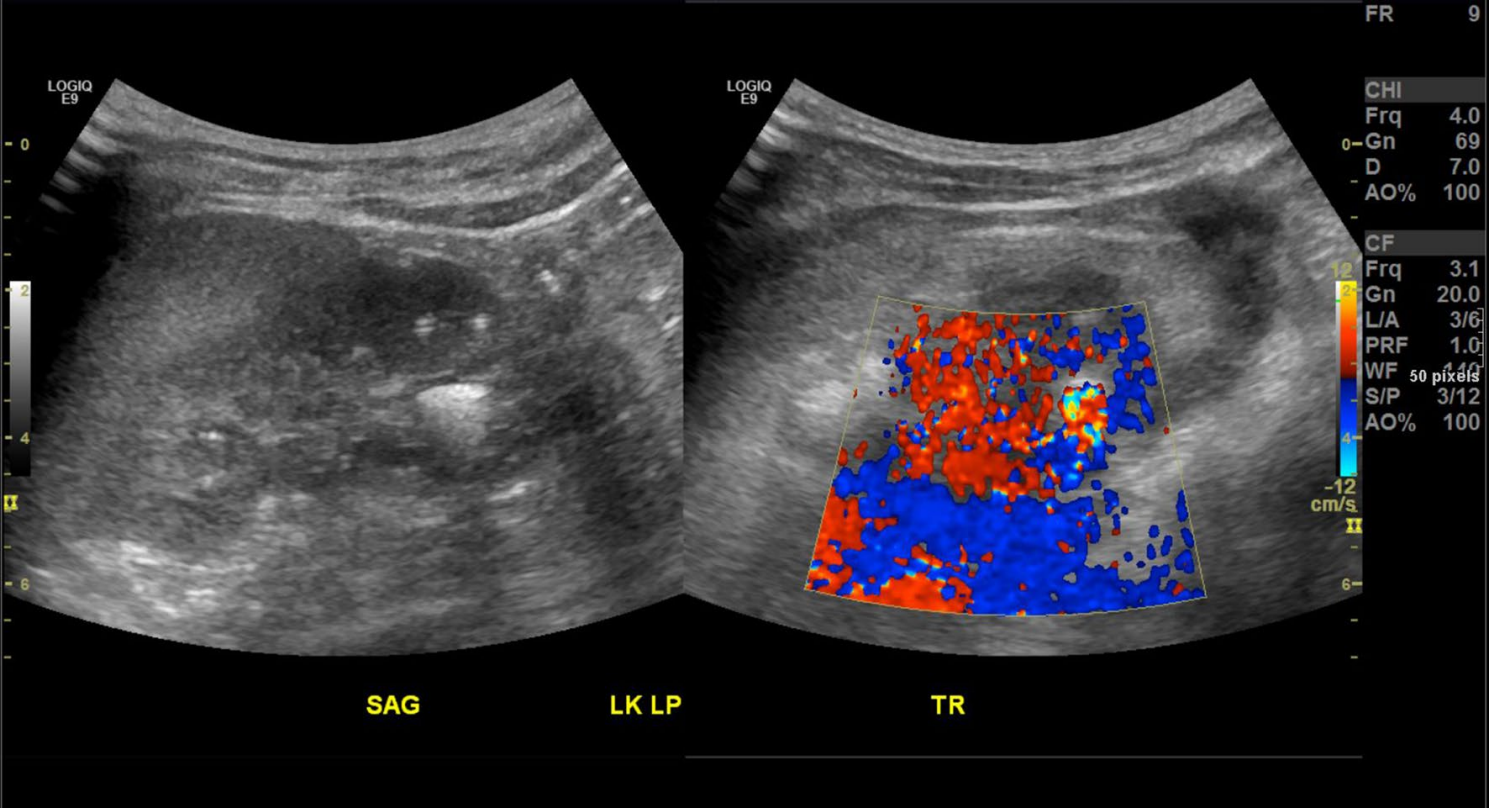
In vivo ultrasound of xanthine urinary stone: stone demonstrates posterior twinkle artifact on color Doppler imaging

On US all stones were echogenic, demonstrated posterior acoustic shadowing, and twinkle artifact with color Doppler imaging on in-vitro analysis (Figs. 4, 5). In-vivo stone analysis performed prior to passage on US demonstrated stones that were echogenic, demonstrated posterior acoustic shadowing, and twinkle artifact (Figs. 6, 7).

**Fig. 8:**
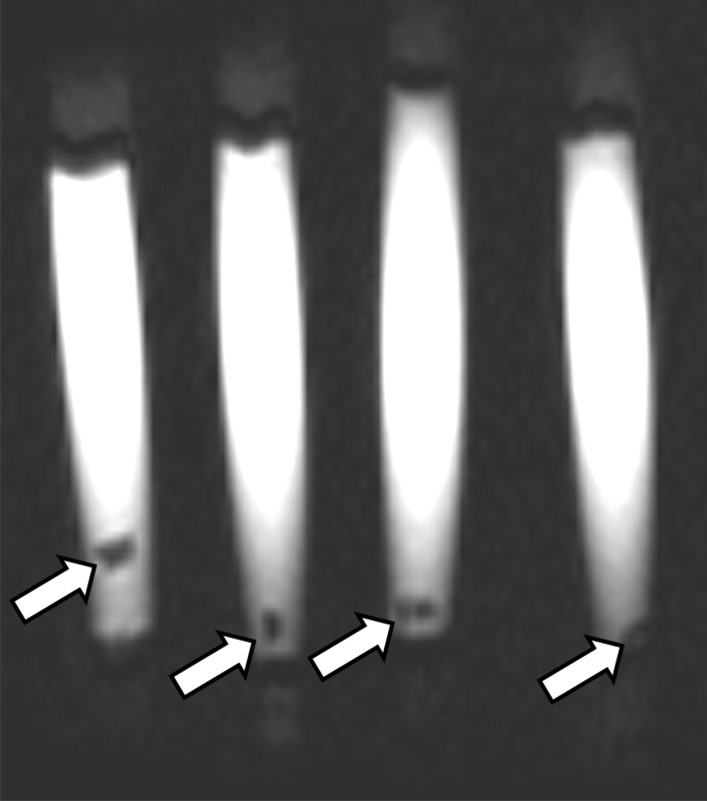
In vitro MRI of xanthine urinary stones: coronal T2-weighted MR image shows stones (arrows) as hypointense signal voids within hyperintense saline-filled syringes

**Fig. 9:**
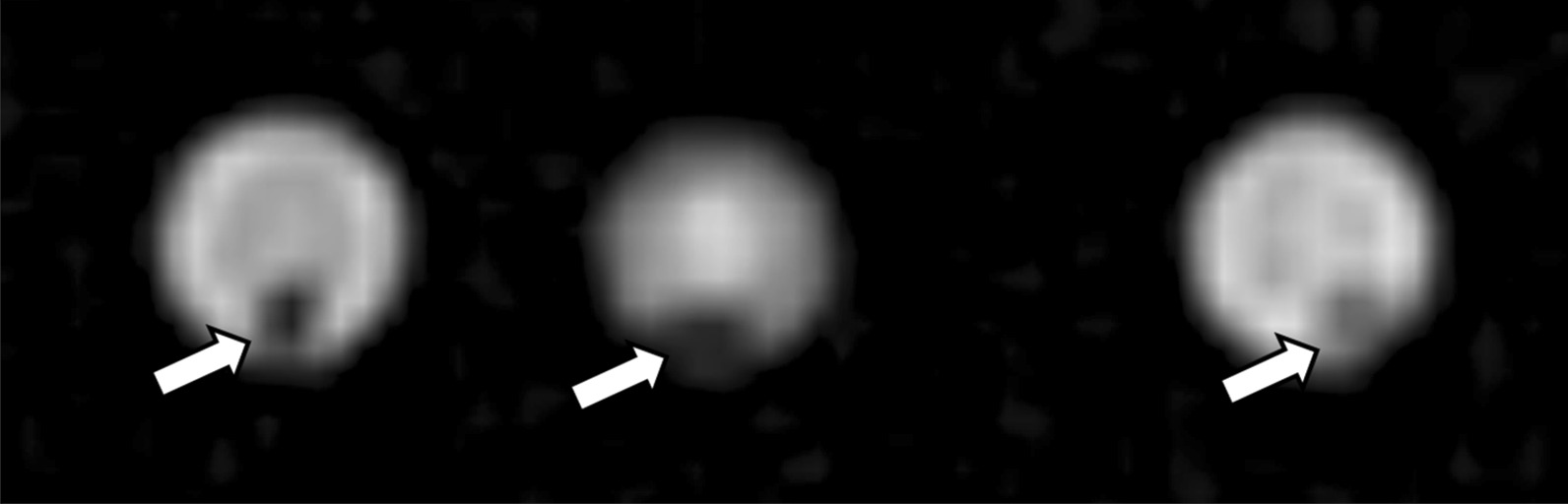
In vitro MRI of xanthine urinary stones: axial UTE-weighted MR image shows stones (arrows) as hypointense signal voids within hyperintense saline-filled syringes

On in-vitro MRI analysis, stones were only visualized as a signal void when imaged in saline-filled syringes, and were not visualized when imaged in air-filled syringes on all sequences, including TSE based T2W sequences, UTE sequences and T2/T1-weighted 3D bFFE sequences (Figs. 8, 9). In-vivo MRI imaging was not available.

## Discussion

To the best of the authors’ knowledge, this is the first in-vitro study describing the imaging characteristics of xanthine stones on CT with multiple different energy levels, US and MRI. A previous in-vivo study of xanthine stones using conventional single energy CT showed CT numbers of stones ranging from 276–480 HU [2]. Previous in-vivo US studies have shown xanthine stones to be echogenic with posterior acoustic shadowing, with features identical to other urinary calculi [3]. No previous studies have described the imaging characteristics of xanthine stones on MRI.

On the four different energy levels on CT, in-vitro xanthine stones had an average CT number of 321.4–331.0 HU. There was no significant difference in the measured HU when imaging at different energies (80, 100, 120 and 140 kVp). On the two in-vivo imaging studies performed prior to stone passage xanthine stones had an average CT number of 304–383 HU. These in-vitro and in-vivo numbers are similar to CT numbers reported in the aforementioned in-vivo study of xanthine stones that used conventional single energy CT.

Interestingly, xanthine stones have relatively lower CT numbers than most urinary calculi. For example, reported mean CT numbers for struvite stones are 401–871 HU, cystine stones are 248–1088 HU, calcium oxalate stones are 865–1039 HU, and calcium phosphate stones are 1417 HU [6–8]. Xanthine stones have similar mean CT numbers to uric acid stones, which have reported CT numbers ranging from 270–519 [6, 8]. This may not be surprising given that xanthine and uric acid are part of the same metabolic pathway. The lower density of xanthine stones likely explains the previous literature describing them as radiolucent on radiographs. The lower density makes them more difficult to appreciate on radiographs than other more dense calculi, and stones were likely radiographically occult rather than truly radiolucent.

On in-vitro and in-vivo US, all xanthine stones were echogenic, showed posterior acoustic shadowing, and demonstrated twinkle artifact with color Doppler imaging. These features are identical to other types of urinary stones. Previous in-vivo analyses of xanthine stones have also showed them to be indistinguishable from other urinary stones on US [1–3]. Based on these findings, ultrasound is equally suitable to evaluate xanthine stones as any other type of urinary stone.

Xanthine stones showed no signal on all in-vitro MRI sequences tested, including UTE MRI sequences. Stones are expected to result in signal voids on conventional MRI sequences, but recent studies of UTE imaging have shown signal within other types of urinary calculi on UTE sequences [9, 10]. This has led some to suggest that these sequences might be utilized to evaluate urolithiasis. Our analysis suggests that xanthine stones are unlikely to be well visualized when utilizing the described MRI techniques in a clinical setting, including UTE sequences.

Although xanthine urolithiasis is a rare condition, it may cause recurrent symptoms in patients with Lesch–Nyhan syndrome on allopurinol therapy and in patients with hereditary xanthinuria. Children with Lesch–Nyhan syndrome are developmentally delayed and are often unable to appropriately verbalize their symptoms, making imaging particularly important in the clinical assessment of these patients. Given the recurrent nature of this condition, multiple imaging studies may be needed over the course of a lifetime. While our study examined rare xanthine stones from a single patient, thus perhaps limiting generalizability, we believe that based on the results of this study, xanthine stones are easily detectable on US. Therefore, US may be the first line imaging test in the evaluation of xanthine stones given its lack of ionizing radiation and ability to visualize these stones. Recent developments in UTE MRI sequences have suggested that MRI may provide an additional imaging modality to assess urinary calculi without ionizing radiation. However, our in-vitro analysis suggests that xanthine stones are not easily detectable on MRI, including UTE sequences, and MRI is unlikely to be helpful in the evaluation of xanthine urolithiasis. Unfortunately, no in-vivo MRI imaging of the stones was performed on this patient prior to stone passage. Our analysis suggests that xanthine stones are well visualized on CT. Therefore, judicious use of conventional non-contrast CT may be appropriate in patients with xanthine urolithiasis when ultrasound is inconclusive or insufficient. The risks of radiation exposure should always be considered, especially given the recurrent nature of this condition and the potential need for multiple imaging studies over a lifetime.

A limitation of our study stems from the small size of xanthine stones on in-vitro analysis. Stones smaller than 5 mm in diameter tend to demonstrate lower CT numbers regardless of composition secondary to partial-volume effects. CT numbers in our in-vitro analysis may be lower given the smaller size of the stones studied. However, on in-vivo analysis, prior to stone passage, the stones were as large as 10 mm and had comparable CT numbers to that of our in-vitro analysis. This may perhaps partially negate the effect of volume averaging on our in-vitro findings. Previous MRI studies that were able to demonstrate signal within urinary stones used stones larger than 1 cm, which is larger than the stones evaluated in this study [7, 10, 11]. This small size may have contributed to the lack of signal seen on all MRI sequences tested in our study. Future studies could benefit from analyzing larger stones, if available.

## Conclusions

Xanthine urinary stones are a rare type of urinary calculus that may cause recurrent symptoms requiring numerous imaging tests over a lifetime. In-vitro and in-vivo analyses showed that xanthine stones are easily detectable on US and CT, but failed to demonstrate signal on all MRI sequences tested. CT numbers of xanthine stones did not vary when imaging with different energies. We believe that US should be considered the first line imaging test in the evaluation of xanthine urinary calculi and judicious use of CT is warranted when ultrasound is inconclusive or insufficient and risks of radiation have been considered.

## Supplementary information

**Additional file 1: **In-vitro CT mean and SD values at four different KVP.

## Data Availability

The datasets used and/or analyzed during the current study are available from the corresponding author on reasonable request.

## Abbreviations

CT: Computed tomography
US: Ultrasound
MRI: Magnetic resonance imaging
FOV: Field of view
HU: Hounsfield units
T2W: T2-weighted
TSE sequences: Turbo spin echo
bFFE: Multi-slice balanced field echo or balanced steady-state free precession
UTE: 3D stack-of-stars ultra-short-TE
TR: Repetition time
TE: Echo time
